# Development and evaluation of a training program for dialysis nurses – an intervention study

**DOI:** 10.1186/s12995-019-0223-3

**Published:** 2019-02-11

**Authors:** Maren Kersten, Sylvie Vincent-Höper, Heidi Krampitz, Albert Nienhaus

**Affiliations:** 1 Institution for Statutory Accident Insurance and Prevention in the Healthcare and Welfare Services, Pappelallee 33/35/37, 22089 Hamburg, Germany; 2 Center of Applied Sciences of Health, Leuphana University of Lueneburg, Wilschenbrucher Weg 84A, 21335 Lüneburg, Germany; 3 Department of Work and Organizational Psychology, University of Hamburg, Von-Melle-Park 11, 20146 Hamburg, Germany; 4 CC Compass Consulting, Rothenbaumchaussee 30, 20148 Hamburg, Germany; 5 Competence Center for Epidemiology and Health Services Research in Nursing (CVcare), University Medical Center Hamburg-Eppendorf, Bethanien-Höfe 52, 20246 Hamburg, Germany

**Keywords:** Dialysis nurses, Intervention study, Stress and resources, Evaluation, Workplace health promotion

## Abstract

**Background:**

A dialysis nurse’s work is complex and demanding. Based on the results of a systematic review and a survey study, we developed a health-promoting intervention for dialysis nurses. The aim of this study is to evaluate this intervention.

**Methods:**

Before the intervention, the dialysis facilities were surveyed, and an analysis workshop was conducted. The intervention incorporated activities at the individual and organizational levels and included three half-day training sessions for dialysis nurses. The evaluation was based on pre-post-follow-up data from the intervention group (*N* = 33) and pre-post data from the control group (*N* = 44) gathered using validated scales. The measurement of change was conducted using repeated measures analysis of variance (ANOVA) and t-tests.

**Results:**

In the intervention group, we found small to medium effect sizes for all measures. However, only sense of community and burnout improved significantly between the pre- and post-tests. Compared to the control group, sense of community increased significantly only in the intervention group. This strengthens the result that the intervention had a particular effect on enhancing the sense of community.

**Conclusions:**

The systematically developed intervention for dialysis nurses offers a promising approach for workplace health promotion in the dialysis setting.

## Background

The ongoing demographic shift and upcoming shortages of professional and managerial staff [1] stress the importance of the prevention of psychological disorders in the health care sector. Health care organizations face the long-term challenge of maintaining qualified, efficiently working, and healthy employees. Thus, one of the primary endeavours for organizations should be to establish health-promoting work environments for their employees.

Dialysis nurses face both positive and critical work characteristics. Positive aspects of the work include that dialysis nurses perceive their work as meaningful, are interested in professional knowledge, and appreciate the freedom to make their own judgements [2]. Furthermore, they experience opportunities to care for other people and value their job security [3]. However, several critical aspects in dialysis work have also been described in the literature. For example, dialysis nurses experience high pressure [4–7] and perceive a lack of involvement in decision-making processes [2, 8]. In addition, stressful work characteristics include repetitive tasks, fear of diseases that are transmitted by blood [2], and underpayment [3]. Because of this special combination of stressors and resources, dialysis work is of particular concern to researchers. To assess the work characteristics that employees face in dialysis work, Böhmert et al. [9] conducted a review of the existing literature, which revealed that the most recent German study on this issue was published 22 years ago. Because the recent findings were lacking, Kersten and colleagues [10] conducted a survey study in 20 different dialysis units to assess psychosocial stressors and resources in dialysis work and compared the results with other health care professions, such as inpatient care (of the elderly). The findings from this study suggest that employees working in dialysis units perceive their work as meaningful. However, they also experience stress – not primarily because of high demands but rather because they perceive a lack of resources, such as feedback, influence at work, or opportunities for development [10]. Therefore, another study focused on the examination of differential effects of resources on employee well-being. In particular, Kersten et al. investigated the buffering effect of the resources feedback and influence at work on the relationship between quantitative demands and stress symptoms [11]. Their results showed no buffering effect of influence at work. However, feedback buffered the effect of quantitative demands on cognitive stress symptoms such that the positive effect of quantitative demands on cognitive stress symptoms was weaker when feedback was high [11].

Based on the results of Böhmert et al.’s [9] review and Kersten et al.’s [10, 11] empirical support, an in-house training program was developed that was tailored to the needs of dialysis work. The key role of resources is in in line with the conservation of resources (COR) theory [12] which states that individuals seek to obtain, retain, foster, and protect resources. According to Kröll et al. [13], stress management interventions help to protect employees’ resources.

Thus, the intervention focuses on fostering resources in dialysis facilities to improve mental health. Based on previous analyses of the target group [9, 10], we focus on social resources that are relevant in team contexts with a high level of interdependency and on individual resources helping to cope with a potentially stressful situation. Following the WHO definition of health [14], many authors acknowledge that well-being includes both (the absence of) negative and positive aspects [15–17]. According to Wright, Emich and Klotz [18], well-being is a multidimensional construct which comprises aspects of impaired well-being (e.g., indicators of exhaustion) and positive states of mind (e.g., job satisfaction). In this study, we draw upon this holistic definition of health and assess effects on both negative and positive aspects of mental health to obtain a differentiated picture.

The objective of the present study is to evaluate the effects of the intervention on different social resources (e.g. social support, sense of community, feedback), individual resources (self-efficacy, health awareness, coping), and positive and negative indicators of employee well-being (e.g., burnout, cognitive stress symptoms, job satisfaction).

Numerous interventions to promote employees’ health and well-being have been implemented by health insurances and institutions for statutory accident insurance and prevention [19]. Several meta-analyses [13, 20, 21] have found small to moderate positive effects of stress management interventions on mental health. There are a number of stress-related intervention studies in the work setting, but most such interventions focus on the individual level (e.g., stress management training [21, 22]). Participants learn to address challenges and stressors differently and, at best, to use resources more efficiently to enhance well-being. In contrast, organizational-level interventions focus on changes in work characteristics and the work environment rather than on cognitive, emotional, or behavioural changes at the individual level. The objective of organizational-level interventions is to redesign work to promote well-being.

Studies have shown that a combination of individual-level and organizational-level interventions have the strongest and longest-lasting effects [23]. In their meta-analysis, Bamberg and Busch [24] concluded that isolated individual- and organizational-level interventions have very few effects. High-quality studies evaluating organizational-level interventions are scarce [24]. Evidence suggests that it is necessary to analyse the situation beforehand to infer approaches and enable a structured process [25]. Kaluza’s meta-analysis of interventions, including primary prevention strategies, showed short-term effects of stress management trainings on negative indicators of well-being, such as anxiety and depressive symptoms [26]. However, according to Kaluza there is a need to investigate medium- and long-term interventional effects using follow-up assessments. Furthermore, Kaluza notes that there is a need to incorporate coping processes when developing stress management trainings [26].

Considering these recommendations, an important characteristic of the present study is that we followed a systematic approach for developing a specific intervention for dialyses nurses. A review regarding main stressors and resources prevalent in dialysis work was conducted as a first step [9]. Based on this review, we identified working conditions in the dialysis setting that are key to health. In a comprehensive study with a sample of employees working in dialysis units, we investigated these factors using validated measures [10, 11]. Drawing on the results of this study, we developed a dialysis-specific in-house training program. In this intervention, we focused on fostering resources to enable dialysis nurses to cope efficiently with the demands at work.

The intervention is theoretically based on the work psychological model of stress [24]. This model is an extension of the traditional stress-strain concept [27] and Lazarus’ [28, 29] transactional model of stress The main characteristic of this model is that the effects of both individual- and organizational-level stressors and resources on well-being are considered. In addition, the model incorporates the intervening role of coping processes following Kaluza’s [26] call to include coping strategies in stress management training.

We suggest that an intervention comprising both organizational- and individual-level aspects (including coping strategies) will have positive effects on dialysis nurses’ well-being.

There is a lack of research on evaluating interventions in the health care sector [30]. To date, only one study among employees working in inpatient care of the elderly [31] integrated individual- and organizational-level interventions and analysed the status quo prior to the intervention, following Nielsens’ [25] recommendations. To the best of our knowledge, there have not been any intervention studies on the provision of work-related resources and the reduction of psychosocial stressors in dialysis work.

The present study aims to close several research gaps and follows recommendations demonstrated in intervention research. First, prior to developing the intervention, we investigated the status quo of resources and stressors in dialysis work [9] to infer approaches from the situation, as claimed by Nielsen and colleagues [25]. Thus, we could target those factors that have been shown to be most relevant for health promotion in this specific context. Second, we followed recommendations [23, 24] to include both individual- and organizational-level aspects in interventions. Third, we followed the recommendations of Kaluza [26] and applied a pre-post-follow-up evaluation design to assess the medium-term effects and to consider the intervening role of coping strategies.

## Methods

### Procedure and study design

A quasi-experimental study was conducted among dialysis nurses from June 2016 to March 2017. Four different dialysis facilities participated in the intervention. One facility each was located in Northern Germany and Central Germany and two facilities were in Southern Germany. The number of staff ranged between 21 to 50 employees. All of the participating facilities belong to the same umbrella organization. In the first step, we asked supervisors working in the facilities to participate in the study. When the supervisors agreed to participate, we asked the works council for their approval. When both the supervisors and the works council agreed to participate, we started with the trainings. In each facility, dialysis nurses voluntary decided to participate. Participants of the training program constituted the intervention group while non-participants constituted the control group. Thus, in each facility we had both an intervention and a control group. Non-participants had the opportunity to participate in the training program after the study was completed. Each facility received in-house face-to-face trainings, that was conducted by one trainer during regular working hours.

The training program ran over a time period of approximately four months. The supervisor training preceded the training program of the dialysis nurses. The supervisors had one session with four hours of direct contact with the trainer. Because the supervisor training was intended to support the implementation of the intervention for the dialysis nurses, we did not provide a survey for the supervisors to evaluate the supervisor training on its own. The dialysis nurses had three sessions with a group size between nine and ten participants and with twelve hours of direct contact with the trainer in total. To avoid that different approaches were applied in the multiple groups, both the supervisor training and dialysis nurses’ training program were conducted by the same trainer.

The dialysis nurses in the intervention group were surveyed at three points in time using validated scales. The first measurement point was directly before the training program, the second was directly afterward, and the third was six weeks after the training program was completed. The scales used in the questionnaire were matched with the content and the objectives of the training program. The dialysis nurses in the control group were surveyed before and after the training program. We refrained from following the control group over time and surveying it at a third point in time six weeks after the training program because the response rate was low, at 35%, and they showed less compliance. We offered no incentives for participating in the survey.

**Fig. 1:**
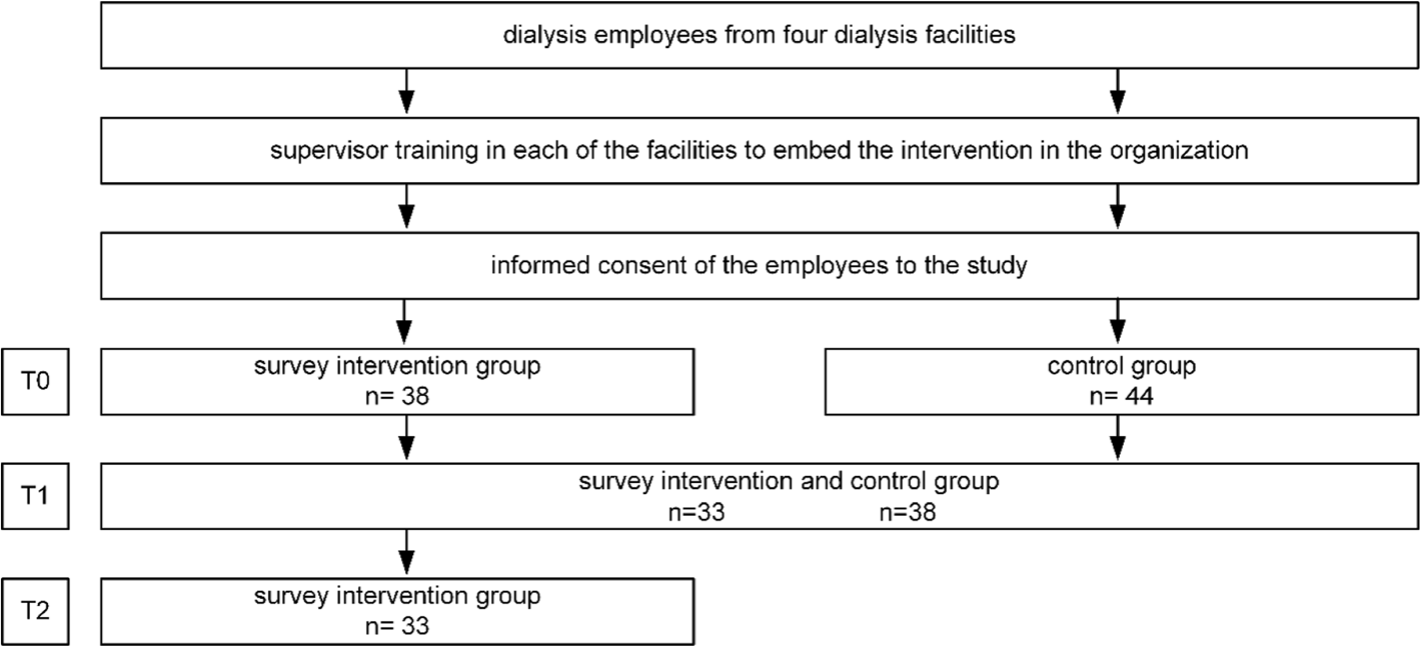
Study design

Figure 1 visualizes the intervention design and the evaluation process with the different measurement points.

### Participants

The intervention included both a training program for dialysis nurses and training for supervisors. The target group of the training program were dialysis nurses who care for patients in dialysis facilities. In the intervention group, 38 nurses in four groups (between nine to ten participants) attended the training program and were surveyed. The survey responses of 33 dialysis nurses could be matched over time. Participants in the supervisor training were each one nursing manager, one medical and one administration director in the four facilities, thus 12 supervisors in total.

#### Sociodemographic characteristics

As shown in Table 1, a total of 33 dialysis nurses in the intervention group and 44 nurses in the control group participated in this study. The sociodemographic characteristics of the intervention group and the control group (e.g., gender, age, type of occupation, type of employment, and duration of employment) were compared (Table 1) and no significant differences were found.

**Table 1:** Sociodemographic characteristics

Sociodemographic characteristics	Intervention group (*N* = 33)	Control group (*N* = 44)	Mann-Whitney U	Χ^2^*(df)*	p
	%	%			
Age			661.5		.458
21 to 30 years	6.1	2.3			
31 to 40 years	6.1	15.9			
41 to 50 years	27.3	29.5			
≥ 51	60.5	52.3			
Gender				.526 *(1)*	.468
Male	12.1	18.2			
Female	87.9	81.8			
Type of occupation				4.252 *(3)*	.236
Certified nurses	87.9	90.9			
Trained nurses	–	2.3			
Nurses in training	–	2.3			
Other nurses (e.g. nurses’ aides, nursing assistants)	12.1	4.5			
Type of employment			682		.612
Full-time > 35 h	48.5	45.5			
Part-time 15 to 35 h	48.5	45.5			
Part-time < 15 h	3	9			
Duration of employment			613		.199
0–5 years	6.1	13.6			
6–10 years	12.1	13.6			
11–15 years	6.1	9.2			
16–20 years	12.1	13.6			
> 20 years	63.6	50			

#### Intervention: Dialysis-specific training program

The content of the training program is shown in the lower section of Table 2. Basically, the program involved stress management through healthy behaviour, health-promoting design of work characteristics, and the identification and enhancement of resources.

**Table 2:** Contents and methods of the intervention

	Objectives	Contents	Methods
1. Information for works council and supervisors	• creating transparency and orientation • ensuring the works councils and supervisors’ support for learning process	• causes for implementing trainings • course of the training • supporting framework conditions	Trainer gives a flip chart presentation and participants engage in a group discussion (plenum)
2. Training of supervisors	• sensitization and information of supervisors • role of leadership for process of implementation • opportunities to ensure knowledge transfer	• principles of health-promoting leadership • healthy self-leadership as a prerequisite of adequate leadership behaviour • methods and guidance to ensure knowledge transfer	• Trainer hands out an information brochure about “Principles of health-promoting leadership” • Participants engage in a group discussion (plenum) on the principles of health-promoting leadership • Trainer hands out an information brochure “Guidelines for transfer interviews” • Trainer explains how to use the guidelines in the transfer interviews
Element 1 of the training program	• stress management through “healthy” behaviour • health-promoting design of work characteristics • identify and foster resources: ▪ enhancing self-efficacy ▪ developing an individual anti-stress program ▪ learning solution-oriented strategies for day-to-day work ▪ improving mutual appreciation ▪ balancing tension and recovery	• definition of concept: What is stress? • becoming acquainted with the “stress traffic lights” with three levels a) stressors b) own attitude and position c) opportunities to recovery ▪ practical approach on the basis of participants’ examples ▪ development of possible coping strategies ▪ planning and implementation of personal intents ▪ planning and implementation of intents on team−/organizational-level	• Trainer gives a short presentation • Participants engage in a group discussion (plenum) • Trainer asks participants to provide examples of stressful situations in their work ➔ Trainer writes different situations on cards that are stressful to dialysis nurses based on Kaluza’s stress concept. • Participants engage in a group discussion (work in small groups and plenum) to develop coping strategies for stressful situations • Trainer gives a short presentation and participants engage in a group discussion (plenum) to reveal (missing) work-related resources in dialysis work and their function in the stress process with a focus on feedback as a resource • Participants develop an individual stress management program using a worksheet • Trainer gives instructions on a relaxation exercise
Element 2 of the training program			
Element 3 of the training program			• At the end of each training session, the trainer guides the group to specify a work-related demand, for which they aim to find a solution until the next training

*The worksheets were provided during the training. The protocol (including photos of the flip charts) was sent via e-mail after the training

The intervention was accompanied by the following activities, which are elements on the organizational-level, to ensure that the training program was embedded in the facilities’ structures:

- The works council was informed about the training program and was asked for approval before the beginning of the intervention.
- We conducted a half-day training for supervisors to sensitize supervisors and to accomplish openness to change.
- The nursing managers conducted transfer interviews with their employees.
- The participants in the employee training program chose spokespersons who were assigned to report back concerns and approaches to solving problems to the supervisors.

These activities provide the framework for an organizational-level intervention. However, as we applied Kaluza’s [32, 33] concept of stress, the intervention also focuses on training personal competencies (e.g., self-reflection).

Aim of the supervisor training was to sensitize the supervisors to issues of leadership and employee well-being and health awareness. The supervisor training incorporated that the supervisors reflected their leadership behaviours and included an introduction to the concept of health-promoting leadership [34]. Moreover, supervisors were trained on how to conduct transfer interviews with their employees, which aimed to consolidate the intervention’s effects in the facility. The nursing managers conducted transfer interviews with their employees before and after the training program. In this interview, the supervisor seeks to discuss the employee’s goals regarding the training program and determine what support the dialysis nurses need to achieve these goals. The supervisors and dialysis nurses conducted a second one-on-one interview after training was completed and discussed if the employee had achieved their goals.

The contents of the intervention are shown in Table 2.

### Measures

In the present study, we report the results of the three measurement points (T0, T1, T2). Standard sociodemographic variables were assessed at each point in time.

The objectives of the training program (see Table 3) were used to choose adequate target variables that were combined into an evaluation measure.

**Table 3:** Objectives of the training program

*Objectives of the training program*	
• fostering job-related resources (improvement of communication, constructive handling of conflicts)	
• improving team cohesion	
• enhancing self-perception (sensitization to own needs)	
• fostering self-efficacy	
• learning solution-oriented strategies for day-to-day work	
• increasing job satisfaction	
• increasing well-being	

The target variables were operationalized using the following measures: social support, feedback, sense of community, occupational self-efficacy, health awareness, coping, burnout, cognitive stress symptoms, and job satisfaction.

Social support, feedback, sense of community, burnout, cognitive stress symptoms, and job satisfaction were measured using the Copenhagen Psychosocial Questionnaire (COPSOQ) [35] in its German version [36]. All of these items were scored on a five-point Likert scale ranging from 1 (“always”) to 5 (“never/hardly ever”). We assessed social support with four items. A sample item is “How often do you get help and support from your colleagues?”. Cronbach’s alphas of the three measurement points ranged between .74 and .83. Sense of community was measured with three items. A sample item is: “Is there good cooperation between the colleagues at work?”. Cronbach’s alphas ranged between .72 and .80. Feedback was measured with two items asking for feedback from colleagues (“How often do you talk with your colleagues about how well you carry out your work?”) and the supervisor (“How often do you talk with your superior about how well you carry out your work?”). The item-total-correlation ranged between .37 and .42. Burnout was assessed with six items. A sample item is “How often do you feel emotionally exhausted?”. Cronbach’s alphas ranged between .89 and .91. Cognitive stress symptoms were measured with four items. A sample item is “How much of the time during the past 4 weeks have you had problems concentrating?”. Cronbach’s alphas ranged between .85 and .91.

We assessed occupational self-efficacy with six items developed by Rigotti, Schyns & Mohr [37]. A sample item is “I can remain calm when facing difficulties in my job because I can rely on my abilities”. Items were scored on a six-point Likert scale ranging from 1 (“strongly agree”) to 6 (“strongly disagree”).Cronbach’s alphas ranged between .81 and .84.

We used the seven-item subscale taken from the Health-oriented leadership (HoL) scale [38] to assess health awareness. A sample item is “I immediately notice when something is wrong with my health”. Items were scored on a five-point Likert scale ranging from 1 (“completely true”) to 5 (“not true at all”). Cronbach’s alphas ranged between .79 and .85.

A four-item scale taken from the Brief COPE was used to measure active coping and planning [39]. Items were scored on a five-point Likert scale ranging from 1 (“completely true”) to 5 (“not true at all”). A sample item is “I’ve been concentrating my efforts on doing something about the situation.” We used the following instruction to ensure that participants rated their coping efforts: “Think about stress, pressure, and difficulties at work in the past two weeks. What have you been thinking and doing?.” Cronbach’s alphas ranged between .78 and .90.

To enable an easier interpretation of the changes, we recoded several scale values such that higher values represent improvements. Exceptions are burnout and cognitive stress, for which lower values indicate that the intervention was effective.

### Statistical analysis

Data analysis was done using SPSS version 22.0 (SPSS, Inc., Chicago, IL, USA). Means and standard deviations (SD) were calculated for continuous variables, and frequencies and percentages for categorical variables.

The effectiveness of the intervention was examined using repeated measure analysis of variance (ANOVA) in which within subject effects were modelled. Model fit was assessed using residual plots and lack-of-fit tests; the significance level was set to α = 0.05. Since the participants of the control group could not be followed over time, we conducted t-tests for independent samples to compare the means of the intervention group and the control group. The assumptions of the repeated measure ANOVA and the t-test were tested (i.e., assumptions of normally distributed data, sphericity assumption and homogeneity of variance). The effect sizes of the ANOVA are interpreted according to Cohen [40], indicating that a partial η^2^ of .01 can be considered a small effect, a partial η^2^ of .06 can be considered a medium effect, and a partial η^2^ of .14 can be considered a large effect.

## Results

Table 4 displays descriptive statistics (mean values and standard deviations) for the three measurement points of the intervention group as well as the results of the repeated measures ANOVAs with an overview of the effect sizes (partial η^2^). The results indicate that all measures display small to medium effect sizes according to Cohen [40] but the effects were mostly insignificant. We found significantly different mean levels between the measurements only for sense of community (F(2, 64) = 4.592, *p* = .014, partial η^2^ = .125) and burnout (F(2, 64) = 3.757, *p* = .029, partial η^2^ = .105).

**Table 4:** Descriptive statistics, Repeated measures ANOVA, t-tests for independent samples

Variable	Intervention group (N = 33)	Control group (N = 44)	T *(df)*	p	Cohen’s d	Results of repeated measures ANOVA^b^		
	M *(SD)*	M *(SD)*				F	p	partial η^2^
Social support								
T0	3.39 *(.77)*	3.57 *(.79)*	−.998 *(75)*	.161	0.231	2.047	.137	.060
T1	3.49 *(.66)*	3.48 (*.80)*	.057 *(69)*	.478	−0.014			
T2	3.61 *(.60)*	–	–	–	–			
Feedback								
T0^a^	1.97 *(.62)*	2.23 *(.85)*	−1.465 *(74.97)*	.065	0.349	1.438	.245	.043
T1	2.21 *(.85)*	2.23 *(.79)*	−.090 *(68)*	.464	0.024			
T2	2.08 *(.64)*	–	–	–	–			
Sense of community								
T0	3.76 *(.46)*	3.75 *(.62)*	.059 *(75)*	.477	−0.018	**4.592**	**.014**	**.125**
**T1** ^**a**^	**4.04** ***(.41)***	**3.72** ***(.73)***	**2.298** ***(59.39)***	***.013***	−0.541*			
T2	3.90 *(.46)*	–	–	–	–			
Self-efficacy								
T0	4.62 *(.75)*	4.53 *(.65)*	.556 (*74)*	.290	−0.128	.364	.697	.011
T1	4.67 *(.61)*	4.54 *(.83)*	.748 *(69)*	.229	−0.178			
T2	4.72 *(.58)*	–	–	–	–			
Health awareness								
T0^a^	3.67 *(.81)*	3.55 *(.49)*	.697 (*49.12)*	.245	−0.179	1.012	.369	.031
T1^a^	3.76 *(.78)*	3.55 (*.34)*	1.388 *(42.72)*	.086	−0.349			
T2	3.84 *(.70)*	–	–	–	–			
Coping								
T0	3.83 *(.81)*	3.50 *(.81)*	1.771 (*74)*	.041	−0.407	.752	.476	.023
T1	3.74 *(.79)*	3.71 *(.60)*	.152 *(68)*	.440	−0.043			
T2	3.92 *(.57)*	–	–	–	–			
Burnout								
T0	3.38 *(.71)*	3.10 (*.71)*	1.756 (*75)*	.042	−0.158	**3.757**	**.029**	**.105**
T1	3.12 *(.61)*	3.08 *(.64)*	.242 *(69)*	.810	−0.064			
T2	3.24 *(.69)*	–	–	–	–			
Stress								
T0	2.49 *(.82)*	2.52 (*.72)*	−.151 (*75)*	.441	0.039	.651	.525	.020
T1	2.35 *(.68)*	2.35 *(.65)*	−.001 *(69)*	.500	0.000			
T2	2.40 *(.69)*	–	–	–	–			
Job satisfaction								
T0	2.59 *(.38)*	2.59 (*.38)*	−1.298 *(75)*	.099	0.000	2.138	.126	.063
T1^a^	2.70 *(.34)*	2.65 *(.59)*	.498 *(60.10)*	.317	−0.305			
T2	2.67 *(.40)*	–	–	–	–			

^a^These variables showed no homogeneity of variance, thus the T-values were calculated for unequal variances

^b^Intervention Group (*N* = 33)

*medium effect

The post hoc tests revealed significant differences between T0 and T1 in the variables sense of community (−.283, 95%-CI [−.468, −.098], *p* = .004) and burnout (.261, 95%-CI [.050, .471], *p* = .017). The effects indicate a significant increase in sense of community and a significant decrease in burnout between pre-measurement (T0) and post-measurement (T1). The changes between post-measurement and follow-up measurement (T2) were not significant, which shows that the mean values were relatively stable. That means, the mean values did neither decrease to baseline-level nor did they increase significantly between post measurement (T1) and follow-up (T2).

The results of the t-tests for independent samples also shown in Table 4 revealed a number of statistically significant differences in the mean levels of the pre-test (T0) between the intervention and the control group. This restricts the comparability of the two groups. Only one variable, sense of community, which had a similar base level in the two groups, shows a significant higher mean level in the intervention group than in the control group. This strengthens the finding of the ANOVA that the intervention had an effect on sense of community.

After the intervention the dialysis facilities continued their work in different ways.

## Discussion

The aim of this study was to evaluate the effectiveness of an intervention for dialysis nurses that seeks to foster job-related resources and reduce job stressors. Pre-post comparisons reveal improvements in all outcome measures. However, analyses of variance show that the effects are only statistically significant for sense of community, burnout, and job satisfaction. The significant improvement of sense of community is corroborated in the comparison with the control group. Albeit not statistically significant, the partial η^2^ of the other measures indicate small to medium effects [40]. Because the statistical significance is dependent upon sample size, it may be conceivable that small effects were not significant because of the small sample size (*N* = 33), i.e., low power. The comparisons show improvements in the measures between pre-test (T0) and post-test (T1). At follow-up, only some measures indicate further (linear) improvements (e.g., social support and mindfulness). However, in most cases, the gains vanished between post-test and follow-up. However, the baseline levels were generally not reached again.

The improvements between T0 and T1, especially in sense of community, may result from the content of the intervention. In each dialysis facility, the intervention group developed shared goals for improving the work situation. The participants inferred and implemented activities to reach the goals. It seems that this fostered the feeling of being part of a community at work, which strengthened the social cohesion and may thus have enhanced the sense of community.

Having the intervention focus on the individual level could be an explanation for why the effects were not stable over time (T1-T2). Although the intervention also incorporated activities at the organizational level, a change in organizational structures needs more time to become effective.

The effect sizes are rather small as one would expect considering the limited intervention period of four months. Evaluations of such interventions generally find small effects [13, 20, 21, 24, 31]. This may be because changes require time to enable the participants to relearn.

The intervention also aimed to improve coping processes in the medium term, as recommended by Kaluza [26]. The learning and practising of coping strategies require learning opportunities and time. In this study, we found no effects of the intervention on coping processes, likely because the follow-up period of six weeks was too short.

The facilities continued in different ways after the intervention was completed. One facility conducted moderated conflict resolution to a conflict between nursing management and dialysis nurses. Willingness to engage in conflict resolution was a result of the intervention through which the nurses recognized the advantages of a collaborative conflict resolution. Beforehand, the administrative direction and the nursing management failed to resolve the conflict because of the nurses’ distrust. The main issue was to create mutual understanding and disclose mutual expectations. Another facility conducted an additional supervisor workshop to decide on next steps. All facilities aimed to continue the intervention. Through self-reflection, changes in perspective and behaviour, and action plans at the organizational level, the intervention gave the impetus to changes in communication structures. The participants recognized that they must become active to bring about effective changes. Management supported the development of approaches that contribute to the enhancement of resources. Thus, the process is initiated but the task has not yet been completed.

This illustrates that the intervention was an initial step in the continuous improvement process. It may therefore be useful to monitor the facilities in the long term and evaluate the results of their efforts. This approach may support dialysis facilities in creating health-promoting work characteristics that keep their dialysis nurses healthy and motivated at work on a long-term basis. Furthermore, this may contribute to the understanding of effective occupational health intervention contents.

## Limitations

In addition to the advantages of this study, such as the structured approach, there are several limitations that need to be considered when interpreting the results. First, the sample size was small. Including more dialysis facilities was difficult because we aimed to have the same trainer conducting the trainings at the facilities to thus avoid methodological differences. Moreover, we surveyed the control group at only two measurement points, and we did not match the responses from the participants at the different measurement points. We chose this approach because we assumed that this group would otherwise have too low a compliance. Moreover, we refrained from randomized assignment to the intervention group and the control group because participation in the training program was voluntary. Therefore, it is possible that the group assignment was subjected to self-selection bias.

The intervention comprised supervisor training and qualified dialysis nurses. A process evaluation from which we refrained due to limited resources would have been useful to detect confounding effects of the different approaches.

Finally, the findings are based solely on self-reports from the participants. The assessment of objective indicators of employee well-being would complement the evaluation of health-promoting effects.

## Conclusion

This is the first study to evaluate a systematically developed intervention with a preceding analysis phase for dialysis nurses that fosters job-related resources and reduces stressors. The evaluation of the intervention showed that among those dialysis nurses who participated in the intervention, all outcome measures indicated a tendency in the expected direction between pre-test (T0) and post-test (T1), however, the effects were mostly insignificant. The strongest significant improvement could be found for sense of community and burnout directly after the intervention. Thus, the intervention has shown to be effective, at least in the short term. To achieve effects that are stable over time, changes should be embedded in organizational structures and monitored over a longer period of time.

## Abbreviations

BGW: Berufsgenossenschaft für Gesundheitsdienst und Wohlfahrtspflege
COPSOQ: Copenhagen Psychosocial Questionnaire
BGW: Institution for Statutory Accident Insurance and Prevention in the Healthcare and Welfare Services
